# Clinical Characteristics, Long-Term Pharmacokinetics, and Outcomes in Kidney Transplant Recipients from an African Tertiary Centre: A 10-Year Single-Centre Retrospective Review

**DOI:** 10.3390/pharmaceutics18010132

**Published:** 2026-01-21

**Authors:** Sadiq Aliyu Hussaini, Caroline Dickens, Confidence Makgoro, Therese Dix-Peek, Badar Munir, Jeevan Perumala, Simran Patel, Qaiser Goolam, Graham Paget, Bala Waziri, Raquel Duarte

**Affiliations:** 1 Department of Internal Medicine, Faculty of Health Sciences, University of the Witwatersrand, Johannesburg 2193, South Africa; caroline.dickens@wits.ac.za (C.D.); confidence.makgoro@wits.ac.za (C.M.); therese.dix-peek@wits.ac.za (T.D.-P.); badarmunir42@hotmail.com (B.M.); jeevanshine1@gmail.com (J.P.); simy.p12@gmail.com (S.P.); qaisergoolam@icloud.com (Q.G.); graham.paget@wits.ac.za (G.P.); balawaziri@gmail.com (B.W.); raquel.duarte@wits.ac.za (R.D.); 2 Department of Medicine, Ibrahim Badamasi Babangida University, Lapai 911101, Nigeria

**Keywords:** graft survival, patient survival, acute rejection, tacrolimus, trough level

## Abstract

**Background:** Kidney transplantation outcomes in resource-limited settings remain underreported. This 10-year retrospective review examined the clinical characteristics, long-term pharmacokinetics, and outcomes of kidney transplant recipients at a South African public hospital. **Methods:** Data from kidney transplant recipients between January 2012 and December 2022 were analysed. Graft and patient survival were assessed using Kaplan–Meier analysis. Cox proportional hazards models were used to evaluate the associations between clinical and pharmacokinetic variables and outcomes. **Results:** The one- and five-year graft survival rates were 87.9% and 65.6%, respectively. Acute rejection, as confirmed by biopsy, was associated with graft failure (HR, 2.46; *p* = 0.010). Increasing recipient age at transplantation increased the graft failure risk by about 5.0% per year (HR: 1.05, *p* = 0.006). Tacrolimus trough and normalised trough levels were lower in the graft loss group 73% and 93% of the time, respectively, despite similar tacrolimus doses. Whereas achieving optimal tacrolimus concentration did not significantly affect graft survival, maintaining a haemoglobin level >10 g/dL improved the chances of 3-, 4-, and 5-year graft survival (*p*-value, 0.001, 0.001, and <0.001, respectively). Patient survival rates were more favourable than graft survival rates. The 1-year and 5-year patient survival rates were 90.0% and 77.4%, respectively. **Conclusions:** This study offers insights into transplant outcomes in low-resource public health settings. The findings emphasise the impact of rejection and age on the risk of graft failure and the significance of maintaining adequate haemoglobin levels after transplantation. The results also indicate the need for more nuanced and personalised approaches to tacrolimus monitoring in the long-term following transplantation.

## 1. Introduction

Kidney transplantation has emerged as a better treatment option for end-stage kidney disease, offering patients improved quality of life and longevity compared to dialysis [1,2]. The success of kidney transplantation is primarily measured by graft and patient survival rates, which serve as critical indicators of the efficacy of transplantation and long-term outcomes.

Data from low- and middle-income settings have been limited, partly due to the absence of dedicated registries, inconsistent reporting, and/or the inherently low-resource nature of the setting [3,4]. In South Africa, the most recent comprehensive data on dialysis and transplant outcomes was the last publication of the national registry in 1994 [5]. Subsequent outcome and survival data have been primarily centre-specific [5,6,7,8], with reports coming from public and private healthcare facilities in Gauteng and the Western Cape [5,6], and the latest year of the follow-up period reported was 2015 [8]. This gap in outcome data reporting establishes the need for more up-to-date studies to gain a deeper understanding of the current landscape of kidney transplantation in the country’s healthcare system. This will shed more light on the state of kidney transplantation in similar settings and provide insight into areas for improvement.

The introduction of calcineurin inhibitors (CNI), such as tacrolimus and cyclosporine, as cornerstone immunosuppressive agents, has significantly improved short-term graft survival in solid organ transplantation, particularly in the first year post-transplantation [9]. However, the management of these immunosuppressive drugs presents a unique challenge, requiring careful monitoring of the pharmacokinetics of these medications and adjustments in their dosages to strike a delicate balance between preventing graft rejection and minimising drug-related toxicity, including long-term adverse effects [10,11,12]. Even with these drugs, long-term graft outcome beyond the first year of transplant has not improved significantly, as the development of chronic allograft nephropathy, sometimes attributed to CNI nephrotoxicity, and other late complications continue to pose significant challenges to long-term graft function and patient survival [9,13]

Given the challenges of kidney transplantations within low-resource settings outlined above and the limited current reports, this study was undertaken to explore the association between clinical characteristics and long-term pharmacokinetics at a public academic hospital in the largest province (Gauteng) of South Africa.

## 2. Methodology

### 2.1. Study Design, Setting, and Participants

This retrospective cohort study reviewed data from kidney transplant recipients at Charlotte Maxeke Johannesburg Academic Hospital (CMJAH) between January 2012 and December 2022. CMJAH is a public, accredited tertiary hospital with a 1088-bed capacity located in Johannesburg, Gauteng Province, South Africa. It offers a wide range of services, including secondary and some highly specialised quaternary services. It also serves both as a referral centre for other hospitals in the Gauteng province and as the main teaching hospital of the University of the Witwatersrand, Johannesburg, South Africa. Johannesburg and Gauteng are the largest city and smallest province in South Africa, respectively. The estimated population of Johannesburg in 2021 was around 5.9 million, with the main ethnicities being: black African 76.4%, White 12.3%, mixed/coloured 5.6% and Indian/Asian 4.9% [14]. The Human Research Ethics Committee-Medical (HREC-Medical) of the University of the Witwatersrand, Johannesburg, South Africa, provided ethical approval for this study (HREC-Medical number: M220339). Patients aged < 18 years at the time of transplantation were excluded from the analysis.

Demographic data collected included sex, age, race, and body height. Baseline characteristics included smoking and alcohol use (pre- and post-transplant), primary kidney disease, type of induction, and donor information (age, sex, and race, where available). Transplant-related data were collected at specific time points if recorded: days 1–14 post-transplant, and months 1, 2, 3, 6, 9, 12, 18, 24, 30, 36, 42, 48, 54, and 60. Data collected included immunosuppression regimen: Calcineurin inhibitor (CNI) daily dose (tacrolimus or cyclosporin); CNI trough or 2 h post-dose whole blood concentration; steroid dose; and other adjunct immunosuppressants (e.g., azathioprine, mycophenolic acid) and laboratory parameters such as haemoglobin, serum creatinine, serum bilirubin, liver enzymes, and serum albumin. Biopsy results were reviewed for the diagnosis of rejection and CNI-related nephrotoxicity.

A consecutive series of all available patient files from those transplanted at CMJAH was selected for analysis. Patients aged < 18 years at the time of transplantation were excluded from the study due to the potential confounding effects of differences in physiology, pharmacokinetics, and duration of underlying pathology on the clinical and outcome variables. Patients transplanted at a different centre and later transferred to and cared for in CMJAH were also excluded.

Concentrations where the CNI dose was taken alongside drugs that interact with calcineurin inhibitors, administered orally or intravenously, and/or taken for two weeks or less were omitted (not captured). Concentrations of tacrolimus taken with Amlodipine, Carvedilol, or Verapamil/Diltiazem were recorded as such. Concomitant use with proton pump inhibitors, prophylactic medications, and non-protease inhibitor HIV drugs was not marked. Additional details on our method for drugs with drug–drug interactions with tacrolimus, later referred to as “co-medication”, can be found in the Supplementary Information Files.

### 2.2. Outcome Definitions

We defined acute CNI-induced nephrotoxicity as a rise in serum creatinine (SCr) above 140 μmol/L, resulting in lowering of the CNI dose, discontinuation of the CNI, or switching to an alternate CNI, followed by a reduction in SCr after these changes in the absence of other explanatory causes such as infection or acute rejection. Post-transplant hypertension was defined as elevated blood pressure (BP) above 130/80 mmHg on more than two records 24 h apart or administration of BP-lowering medications that continued beyond the last follow-up. Post-transplant diabetes mellitus was defined by clinician initiation and continuation of new glucose-lowering therapy (insulin or oral glucose-lowering agents) due to sustained elevated fasting blood glucose, 2 h postprandial glucose, or HbA1c levels within the first year of transplantation. We defined overall graft loss as a return to dialysis or death with a functional graft. We defined the optimal tacrolimus concentration in the first month post-transplantation as a trough concentration of 8–15 ng/mL. Subsequently, maintenance of optimal concentration was considered to have occurred when a trough level of 5–10 ng/mL was recorded in more than 33% of measurements. We categorised maintaining a haemoglobin level >10 g/dL (considered optimal) in more than 75% of measurements versus less than 75% of the measurements. Pharmacokinetic analysis was limited to tacrolimus due to the small number of patients on cyclosporine.

### 2.3. Synopsis of Transplant Care Protocol

#### 2.3.1. Risk Stratification and Immunosuppressive Agents

Patients are classified as high risk if they have had a previous transplant, have panel reactive antibody (PRA) by Luminex > 30%, are HIV-positive recipients, or are children. They are classified as low risk if they are first transplant recipients, are adults, are HIV-negative recipients, or have PRA by Luminex < 30%.

High-risk patients are induced with Antithymocyte globulin (ATG), given as 1.5 mg/kg daily for 5 days, with intravenous Hydrocortisone sodium succinate 100 mg premedication and intravenous Methylprednisolone sodium succinate 500 mg in theatre on day 0.

Low-risk patients are induced with Basiliximab 20 mg IV on day 0 and 4 and intravenous Methylprednisolone sodium succinate 500 mg in theatre on day 0. Both high and low risk recipients are given oral Prednisone 60 mg daily on day 1 and weaned by 5 mg per week to the lowest dose of 5 mg per day.

Tacrolimus is provided by the institution in oral form, taken twice daily. Patients are advised to take it on an empty stomach or two to three hours after food. In total, 0.15 mg/kg of tacrolimus is given in two divided doses, with a level of 10 to 15 ng/mL (before 2019) and 10 to 12 ng/mL (from 2019 onwards) as a target for the first three months, then 5 to 8 ng/mL afterwards in both high- and low-risk recipients. This has not changed during the study period. Low-risk recipients are given tacrolimus (as for high-risk) or Cyclosporine (Neoral), with a loading dose of 8 mg/kg followed by 4 mg/kg twice daily orally thereafter, targeting a C2 level of 1000 ng/mL for the first three months and 600 to 800 ng/mL subsequently. These concentrations serve as general guides, but dose adjustments and whole-blood concentration targets largely depend on the discretion of managing nephrologists and clinical scenarios. Mycophenolate mofetil 1–1.5 g twice daily orally or Azathioprine 2 mg/kg daily orally are used in conjunction with CNI. mTOR inhibitors (Sirolimus and Everolimus) were not used either for induction or in conjunction with calcineurin inhibitors in our cohort.

#### 2.3.2. Infection Prophylaxis

Recipients are prophylactically given Cotrimoxazole equivalent to 800/160 mg per day for 6 months, or Dapsone 100 mg per day (if Sulphur allergic). Isoniazid 200 mg per day if <50 kg or 300 mg per day if >50 kg, with 25 mg pyridoxine per day for 6 months. Valganciclovir 900 mg per day (if GFR 40–60 mL/min), 450 mg per day (if GFR 20–40 mL/min) and 450 mg alternate days (if GFR < 20 mL/min) for three months except where both donor and recipient are negative for cytomegalovirus. An oral proton pump inhibitor, equivalent dose of omeprazole 20 mg or lansoprazole 30 mg, is given daily. Oral nystatin for mouth rinsing is given for the first month after transplant.

#### 2.3.3. Calcineurin Inhibitors CNI Level Measurement

Since 2007, our institution has used High-Performance Liquid Chromatography-Mass Spectrometry (HPLC-MS/MS) to measure whole-blood concentrations of tacrolimus and cyclosporine. We also participate in LGC Standards’ External Quality Assurance programme, which is the largest scheme for immunosuppressants using liquid chromatography Tandem Mass Spectrometry (LC-MS/MS).

### 2.4. Statistical Analysis

Baseline clinical and demographic characteristics and transplant-related data were described using summary statistics. Variables were assessed for normality using a Shapiro–Wilk test and found to be non-normally distributed. Continuous variables were summarised as medians (with interquartile ranges [IQRs]), and groups were compared using the Mann–Whitney U test. Categorical variables were summarised as frequencies and percentages, and groups were compared using Pearson’s chi-squared or Fisher’s exact test.

The primary outcomes were time to death and time to overall graft survival. The survival probabilities at 1, 2, 3, 4, and 5 years were estimated using the Kaplan–Meier method, and the associations between risk factors and primary outcomes (time to death or time to death-censored graft failure) were analysed using Cox proportional hazard models. Variables included in survival models were chosen a priori based on their clinical relevance (recipient age at transplant, sex, reaching and maintaining optimal CNI whole blood concentration, maintaining a haemoglobin level >10 g/dL in more than 75% of measurements, use of co-medications in at least 75% of measurements, pre-transplant hypertension, pre- and post-transplant diabetes status, and rejection episodes). Statistical significance was set at *p* < 0.05. All analyses were conducted using Stata software version 18.0 (StataCorp, College Station, TX, USA) and the stset suite of commands.

## 3. Results

### 3.1. Demographic and Clinical Characteristics

A total of 194 adult kidney transplantations were performed between January 2012 and December 2022. Five (5) patients as were transferred to other centres for continued care and thus were censored. Table 1 shows the baseline clinical and demographic characteristics of the cohort, comparing the groups based on graft survival of at least 5 years. The cohorts had similar characteristics in both groups, except for the median age at transplantation, evidence of rejection episodes, and pre-transplant diabetes mellitus (Table 1). The majority of patients were administered tacrolimus as the main immunosuppressant medication (Table 1); as a result, further pharmacokinetic analysis was limited to the tacrolimus pharmacokinetic profile.

### 3.2. Graft and Patient Survival Analysis

**Figure 1:**
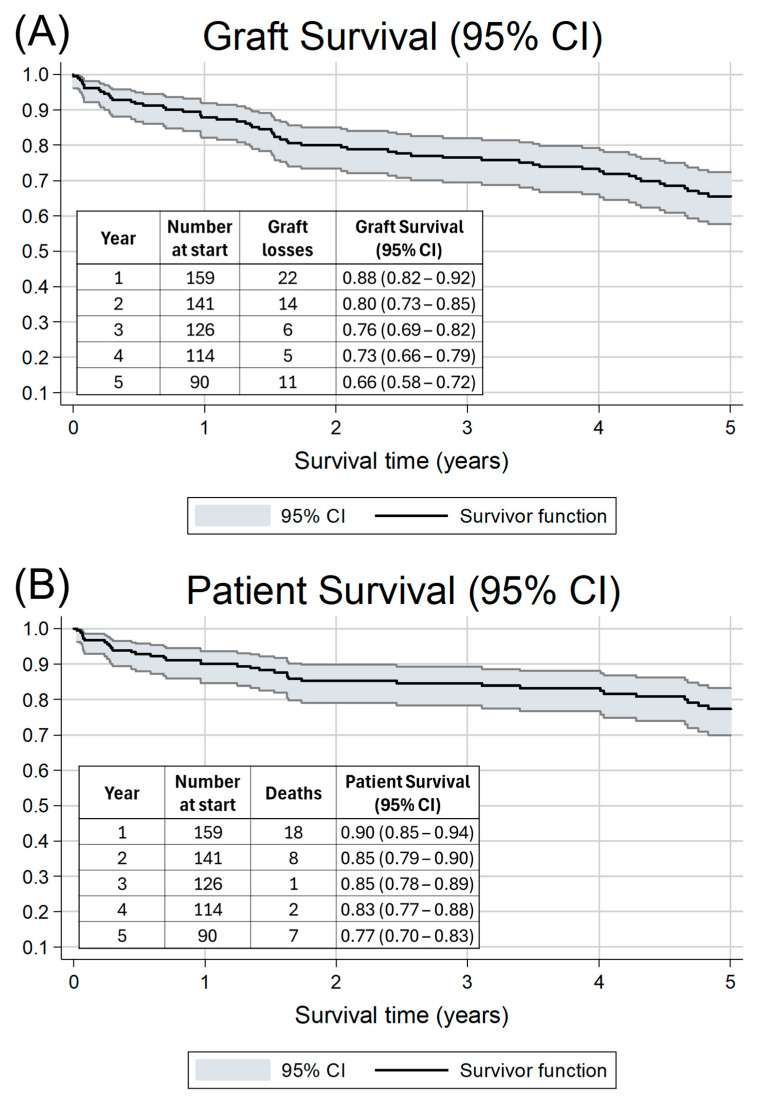
Kaplan–Meier analyses of 5-year (**A**) graft and (**B**) patient survival indicating both the survival function and 95% confidence interval (CI). Inset tables show the number at risk at the start of each year, the number of losses per year, and the survival rate with 95% CI.

Figure 1A,B show the graft and patient survival rates over 60 months (5 years). At the last censorship period, graft survival was 65.56% (57.72—72.29), while patient survival was 77.41% (69.88–83.29). An event of acute rejection, as confirmed by biopsy, was found to be independently and significantly associated with graft failure (HR: 2.16, *p*-value: 0.006, 95% CI: 1.25–3.74) and remained significant even after adjusting for other important variables (Table 2). Sex was not associated with graft survival; however, a 3.4% increased chance of graft failure was observed for every 1-year increase in age at transplant (HR: 1.03, *p*-value: 0.010, 95% CI: 1.01–1.06). This association also remained significant after adjusting for confounding factors. When examining overall patient survival, only age at transplantation was significantly associated with patient loss in both the unadjusted and adjusted models (Table 2).

### 3.3. Graft Loss Profile

A total of 68 recipients lost their grafts within five years, of whom 10 (14.7%) had grafts which had never functioned (primary non-function). Among these, 55 (80.9%) had at least one biopsy. Of those biopsied, 33 (60%) had a diagnosis of either rejection, BK virus nephropathy, or CNI nephrotoxicity. Specifically, 22 (40%) had at least one rejection diagnosis, with 15 (27.3%) experiencing one rejection, 5 (9.1%) experiencing two rejections, and 2 (3.6%) experiencing three rejections. These 22 (40%) cases were characterised by eight episodes of cellular rejection only, seven episodes of antibody-mediated rejection only, and seven episodes with both cellular and antibody-mediated rejection at different times.

Five patients (9.1%) were diagnosed with BK virus infection, and ten (18.2%) showed biopsy features of CNI nephrotoxicity. Additionally, five (9.1%) had both rejection and CNI nephrotoxicity diagnoses, while one (1.8%) recipient had all three diagnoses: rejection, CNI nephrotoxicity, and BK virus nephropathy. Twenty-seven (39.7%) graft losses occurred in recipients who died with a functioning graft. Overall, there were two diagnoses of chronic rejection: one in isolation and the other following two prior diagnoses of acute rejection. In contrast, in the graft-surviving group, 33 (36.3%) had at least a diagnosis of rejection, CNI nephrotoxicity, or BK virus nephropathy (*p*-value: 0.009). Fifteen (16.5%) had at least one rejection (*p*-value: 0.003); twelve (13.2%) had one rejection (*p*-value: 0.057), three (3.3%) had two rejections, and none had three rejection diagnoses. Additional details can be found in the Supplementary Materials.

### 3.4. Pharmacokinetic Profile of Tacrolimus

**Figure 2:**
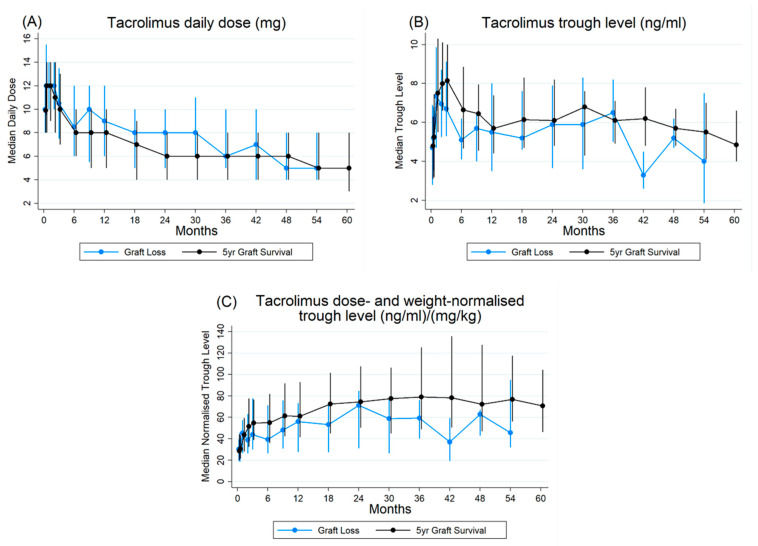
Median tacrolimus (**A**) daily dose (mg), (**B**) trough level (ng/mL), and (**C**) dose- and weight-normalised trough levels (ng/mL)/(mg/kg) for participants who lost their graft within 5 years of transplant and those whose graft survived more than 5 years post-transplant. Timepoints plotted in the first year post-transplant are day 7, day 14, month 1, month 2, month 3, month 6, month 9, and month 12. Thereafter 6 monthly timepoints are plotted up to month 60 (5 years post-transplant). Error bars indicate interquartile ranges.

Following the assessment of graft survival rates over 60 months (Figure 1), we further explored the pharmacokinetic profiles of the primary immunosuppressant therapy, tacrolimus, to understand its impact on these outcomes. Analysis of immunosuppressant therapy revealed that the median daily tacrolimus dose varied across different post-transplantation time points (Figure 2; Supplementary Table S1). It also showed that doses were high in the first year post-transplantation and were subsequently reduced, remaining relatively constant in subsequent years. There were no significant differences in the median doses between recipients whose grafts survived and those who lost their grafts, although, in general, those with graft loss had slightly higher median doses. (Figure 2; Supplementary Table S1).

The median trough tacrolimus levels also fluctuated over time. Trough levels were higher in the first six months post-transplant and lower at subsequent time points. Interestingly, the median troughs were generally lower (73% of the time) in the graft loss group, reaching statistical significance at three time points: months 2, 6, and 42 (Figure 2, Supplementary Table S2).

To account for individual variability in drug metabolism relative to body composition, we calculated dose- and weight-adjusted tacrolimus trough levels to provide a more standardised measure of drug exposure across patients. The median values of the adjusted levels are presented in Figure 2 (Supplementary Table S3). Normalised trough levels were lower in participants who lost their grafts compared to those whose grafts survived 93% of the time, reaching significance at months 6, 18, and 42 (Figure 2, Supplementary Table S3).

### 3.5. Pharmacokinetics and Graft Survival

Achieving an optimal tacrolimus concentration in the first month post-transplant was not associated with graft survival in the first year post-transplant, nor was maintaining an optimal tacrolimus concentration in more than 33% of measurements within the first year (Table 3). Similarly, there was no difference in the hazard ratios between those whose grafts survived and those who lost their grafts when examining the maintenance of optimal trough concentrations for the 5-year survival period (Table 3). This remains true even after adjusting for age at transplantation and maintenance of optimal haemoglobin levels. Conversely, maintaining a haemoglobin level > 10 g/dL in more than 75% of the measurements improved the chances of 3-year, 4-year, and 5-year graft survival (at 36 months: HR; 0.31, *p*-value; 0.004, 95% CI, 0.14–0.69; at 48 months HR; 0.29, *p*-value; 0.001, 95% CI, 0.14–0.59; at 60 months HR; 0.22, *p*-value; <0.001; 95% CI, 0.12–0.43) (Table 3).

Co-medication use was omitted at months 1 and 48, as no patients who lost their grafts in these time intervals were on co-medications at least 75% of the time, and thus models failed to converge.

## 4. Discussion

### 4.1. Graft Survival

The study revealed graft survival rates of 87.9% and 65.6% at 1- and 5-years post-transplantation, respectively. These rates are lower than, but comparable to, those reported in other studies, such as those from the Wits Donald Gordon Medical Center, a private setting in the Gauteng province, South Africa, with 91.3% and 79.1% 1- and 5-year graft survival, respectively [5], and the Groote Schuur Hospital (GSH), a public setting in the Western Cape province, South Africa, with 89.4% and 80.0%, respectively [8]. These outcomes are also similar to those reported from other resource-limited settings, including a cohort of 47 living–related donor transplants in Nigeria (95.3% and 60.7%) [15] and 70.0% 1-year survival in 1356 living–related donor transplants in Mexico [16], as well as 87.0% and 64.0% 1- and 5-year graft survival in another combined Mexican cohort of 326 recipients [17]. The lower graft survival rate compared to other South African cohorts may be attributed to the larger proportion of living donors in those studies (33.3% at GSH, 40% at WDGMC, versus 11.8% in our cohort) [5,8]. Furthermore, the GSH investigators reported death-censored graft loss, whereas for the WDGMC (private centre), socioeconomic factors—such as challenges in long-term follow-up and socioeconomic barriers affecting medication adherence—are likely to be more favourable, as these patients attend a private healthcare facility compared to those attending public health facilities. As demonstrated by Gordon et al. (2020) [15], in South Africa, like most other low- and middle-income countries, the least advantaged individuals with low socioeconomic levels access the public health sector, while high-socioeconomic-status individuals, most of whom have health insurance coverage, mainly access private hospitals. Two previous studies from our setting shed additional light on the nature of patients with chronic kidney disease and the role that socioeconomic status may play. Nqebele et al. evaluated the influence of socioeconomic status and demonstrated a lower risk of kidney disease in individuals with a high socioeconomic status [16]. Another study evaluated the clinical and demographic characteristics of black ethnicity patients with advanced chronic kidney disease and found that more than a third (35%) of the patients were either unemployed or domestic workers, and over 30% had at most a primary school level of education [14].

Our study also included a high proportion of individuals of Black ethnicity. Multiple studies have highlighted low graft survival and poor kidney transplant outcomes in this patient population [5,17,18,19]. Factors contributing to this include: relatively lower socioeconomic status, lower rates of living–related donation, higher rates of rejection, and persistence of hypertension post-transplantation [5]. Genetic mutations in the *APOL1* gene in living donors of African ancestry, higher rates of HLA mismatching, and genetic variants that contribute to an extensive and highly variable tacrolimus metabolism may also play a role in negative graft outcomes after transplantation [5,19,20,21].

An important finding was the significant association between acute rejection, as confirmed by biopsy, and graft failure. Age at transplantation emerged as a significant factor influencing graft survival, with an increased risk of graft failure with increasing age. Moreover, maintaining haemoglobin levels > 10 g/dL in more than 75% of the measurements improved the survival chances at 36, 48, and 60 months.

In our cohort, 68 recipients experienced graft loss. About 81% had at least one biopsy, and 40% showed rejection, suggesting immunologic injury as a principal cause of graft failure. This proportion parallels major studies showing that alloimmune injury—especially antibody-mediated and chronic rejection—remains the leading determinant of late graft failure [22].

The high biopsy rate strengthens diagnostic confidence, though unbiopsied failures may conceal additional immune injury. Overlapping lesions were frequent, as several grafts showed both rejection and CNI nephrotoxicity or BK virus nephropathy, reflecting the complex interplay between immunosuppression, infection, and alloimmunity.

Death with a functioning graft accounted for 39.7% of losses, comparable to registry data [23] and underscoring the need to consider competing-risk methods when estimating immunologic graft failure. The two cases of chronic rejection, one following multiple acute rejections, shows a continuum from acute to chronic alloimmune injury. The greater rejection frequency among failed versus surviving grafts (*p* < 0.001) reinforces its prognostic importance.

Despite the absence of data on donor-specific antibody, our findings emphasise that immune-mediated processes remain a major, potentially modifiable, cause of graft loss. The convergence of diagnoses creates a difficult and often unresolvable therapeutic paradox, especially in a low-resource setting. For example, reducing CNI dosage to treat BK virus nephropathy risks acute rejection, while maintaining levels exacerbates CNI toxicity—often accelerating the final trajectory to graft failure. These factors further reinforce the requirement for individualised and more scenario-tailored immunosuppression.

### 4.2. Dose, Trough, and Normalised Trough Trends

The analysis of tacrolimus dosing and trough levels revealed some interesting patterns. While median daily doses were generally similar in both groups, trough levels were lower in the graft loss group 73% of the time, and significantly so at months 2, 6, and 42. The dose started high and decreased with increasing time after transplantation. This is consistent with the expected decrease in dose requirements over time after transplantation [24,25]. Explanations offered for this range from improvement in haematocrit and albumin levels to a decrease in steroid doses and metabolic enzyme activity [26].

Tacrolimus trough levels have emerged as the gold standard surrogate for measuring tacrolimus exposure in kidney transplantation [27] and are recommended in the KDIGO 2010 kidney transplant guidelines and the second consensus report of the International Association of Therapeutic Drug Monitoring and Clinical Toxicology (IATDMCT) (2019) [28,29]. Interestingly, achieving optimal tacrolimus concentrations of 10–15 ng/mL in the first month and maintaining levels of 5–10 ng/mL for more than 33% of the time did not significantly impact graft survival in this cohort within the first 60 months. This suggests that although monitoring trough levels has become the established practice, it may not be sufficient for assessing adequate tacrolimus exposure and thus sufficient immunosuppression, indicating the need to develop and evaluate other avenues for dosing and therapeutic drug monitoring [12,30].

The normalised trough concentration (dose- and weight-adjusted) was numerically higher in patients with surviving grafts (93.3% of the time) than in trough level measurements (73% of the time), although statistical significance was reached only at specific time points. This suggests that the dose-adjusted trough level might be a better guide to achieving adequate drug exposure than the present method of aiming to meet target trough levels [12].

Although the above findings may reflect the complexity of post-transplant management, they suggest the need for more nuanced approaches to immunosuppression monitoring, such as the practical and easily implementable approach of monitoring dose-adjusted trough levels instead of simple trough levels. They also emphasise the importance of managing post-transplant comorbidities, such as anaemia. Fortunately, these are factors that can be reasonably well-managed even in resource-limited settings.

### 4.3. Patient Survival

Patient survival rates showed a much brighter outlook than graft survival rates. The 1-year and 5-year patient survival rates were 90.0% and 77.4%, respectively. These figures were comparable to those in similar limited-resource settings, such as Cape Town (*n* = 198; 90.4% graft survival and 83.1% patient survival) [8], WDGMC (*n* = 368; 93.7% graft and 86.1% patient survival) [5], and Mexico (92.0% and 81.0% graft and patient survival, respectively) [31]. Patient survival rates were significantly influenced by age at transplantation.

### 4.4. Limitations

By virtue of its retrospective nature, our study had a limited ability to capture data on variables such as the type of kidney replacement therapy prior to transplantation, episodes of infection, adherence to treatment, socioeconomic statuses, and markers for delineation of immunological contribution. However, we captured other data, such as antibody induction and time-varying haemoglobin levels, and applied normalisation that may help mitigate the absence of these and other contributing variables. Nearly 40% of graft losses were due to patient death while the graft was still functioning. This is a significant proportion and emphasises that patient survival is an important competing risk in graft-failure analyses. This phenomenon can dilute the relative contribution of immunological causes to graft-failure when both are analysed via Kaplan–Meier. It is therefore recommended in future studies—where accurate delineation can be made—to use competing-risk methodology to appropriately partition the incidence of immunological graft loss from death with a functional graft and nonimmunological graft loss.

## 5. Conclusions

Our study provides insights into graft survival rates and factors influencing outcomes in kidney transplant recipients from an African public health setting. These findings highlight the impact of acute rejection and age at transplantation on graft failure risk, while emphasising the importance of maintaining adequate haemoglobin levels. While tacrolimus dosing and trough levels showed expected patterns aligned with guidelines, optimal concentrations did not significantly impact graft survival, suggesting the need for more nuanced approaches, such as monitoring dose-adjusted trough levels. Our findings demonstrate the complex interplay of factors influencing graft and patient survival, including immunosuppression management and the presence of comorbidities. Further research is needed to explore alternative methods for assessing tacrolimus exposure, integrate pharmacogenetics, and optimise long-term outcomes in low-resource settings.

## Figures and Tables

**Table 1:** Baseline clinical and demographic characteristics of the cohort.

	Overall	5-Year Graft Survival	Graft Loss	*p*-Value ^+^	Number of Missing Records
	*n* = 194	*n* = 121	*n* = 68		5 ^!^
Age at transplant (Median, IQR)	41.5 (34.0–49.0)	40.0 (34.0–49.0)	45.5 (37.5–51.0)	0.029 ^$,^*	
Males (%)	122 (65.1%)	73 (60.3%)	49 (72.1%)	0.106	
First Kidney transplant (%)	165 (95.9%)	100 (95.2%)	65 (97.0%)	1.000 ^#^	22
Cadaveric Donor (%)	171 (88.1%)	105 (86.8%)	62 (91.2%)	0.365	
Ethnicity (%)		-	-		8
Black	158 (85.0%)	100 (82.6%)	55 (90.2%)	0.466 ^#^	
White	13 (7%)	8 (6.6%)	4 (6.6%)		
Asian	6 (3.2%)	5 (4.1%)	1 (1.6%)		
Mixed	9 (4.8%)	8 (6.6%)	1 (1.6%)		
Antibody induction (%)					19
Antithymocyte globulin (ATG)	91 (52.0%)	50 (47.6%)	40 (60.6%)	0.148 ^#^	
Basiliximab	81 (46.3%)	52 (49.5%)	26 (39.4%)		
Other	3 (1.7%)	3 (2.9%)	0		
Co-medication (%) Yes	120 (64.9%)	77 (64.7%)	42 (67.7%)	0.683	9
Calcineurin type (%)					4
Cyclosporine	5 (2.6%)	1 (0.8%)	2 (3.1%)	0.548 ^#^	
Tacrolimus	146 (76.8%)	94 (77.7%)	49 (76.6%)		
Both (Switch)	39 (20.5%)	26 (21.5%)	13 (20.3%)		
Adjunct immunosuppressant ^££^				0.003 *	
MMF	164 (84.5%)	106 (87.6%)	53 (77.9%)		
MMF/AZT	21 (10.8%)	14 (11.6%)	7 (10.3%)		
Neither MMF/AZT	9 (4.6%)	1 (0.8%)	8 (11.8%)		
Delayed Graft Function (%)	54 (28.7%)	29 (25.0%)	25 (37.3%)	0.078	6
Pre-Transplant Hypertension (%)	160 (94.7%)	104 (93.7%)	52 (98.1%)	0.219	25
Post-Transplant Hypertension (%)	163 (91.1%)	109 (93.2%)	52 (91.2%)	0.649	15
Pre-Transplant Diabetes (%)	4 (2.2%)	0	3 (5.0%)	0.038 *	13
Post-Transplant Diabetes (%)	21 (10.8%)	12 (9.9%)	9 (13.2%)	0.486	
Acute Nephrotoxic event (%)	27 (15.8%)	17 (15.5%)	9 (15.8%)	0.955	23
Rejection episode	37 (20.1%)	15 (13.5%)	22 (32.4%)	0.003 *	10
Primary kidney disease					
Chronic glomerulonephritis	16 (8.3%)	10 (8.3%)	6 (8.8%)	0.219 ^#^	
Diabetic nephropathy	4 (2.1%)	1 (0.8%)	3 (4.4%)		
Hypertension	88 (45.4%)	52 (43.0%)	34 (50.0%)		
Polycystic kidney disease	7 (3.61%)	3 (2.5%)	4 (5.9%)		
Other	6 (3.1)	4 (3.3%)	1 (1.5%)		
Unknown	73 (37.6%)	51 (42.2%)	20 (29.4%)		

^+^ *p*-values calculated using Pearson’s chi-squared test unless otherwise indicated. ^$^ *p*-value calculated using Mann–Whitney test; ^#^ *p*-value calculated using Fisher’s exact test. * *p*-value significant, less than 0.05. ^!^ Censored—transferred to a different centre. Co-medication—concomitant administration with Amlodipine, Carvedilol, verapamil, or diltiazem. ^££^ Adjunct immunosuppressant: MMF; Mycophenolic acid, MMF/AZT; a switch between Mycophenolic acid and Azathioprine, Neither; Neither of Mycophenolic acid or Azathioprine. The significant *p*-value is due to early graft loss before immunosuppressant initiation in grafts that never functioned.

**Table 2:** Hazard ratios for graft and patient survival.

**Graft Survival**				
**Risk factors**	**Unadjusted**		**Adjusted ^#^**	
	**HR (95% CI)**	***p*** **-Value**	**HR (95% CI)**	***p*** **-Value**
Rejection YES (Ref NO)	2.16 (1.25–3.74)	0.006	2.25 (1.13–4.47)	0.021
Age at transplant (years)	1.03 (1.01–1.06)	0.010	1.05 (1.01–1.08)	0.006
Female gender (Ref Male)	0.70 (0.40–1.21)	0.203	-	
Pre-transplant diabetes YES (Ref NO)	3.01 (0.42–21.88)	0.275	-	
Post-transplant diabetes	1.40 (0.69–2.86)	0.350	-	
Pre-transplant hypertension YES (Ref NO)	3.06 (0.42–22.24)	0.268	-	
Delayed graft function	1.29 (0.73–2.27)	0.384	-	
**Patient Survival**				
**Risk Factors**	**Unadjusted**		**Adjusted ^$^**	
	**HR (95% CI)**	***p*** **-Value**	**HR (95% CI)**	***p*** **-Value**
Age at transplant (years)	1.03 (1.00–1.07)	0.050	1.06 (1.02–1.11)	0.005
Female gender (Ref: Male)	0.69 (0.34–1.40)	0.304	-	
Pre-transplant diabetes YES (Ref: NO)	4.66 (0.63–34.42)	0.131	-	
Post-transplant diabetes YES (Ref: NO)	0.45 (0.11–1.87)	0.271	-	
Pre-transplant hypertension YES (Ref: NO)	1.58 (0.21–11.67)	0.654	-	

^#^ Adjusted for rejection, age at transplant, sex, delayed graft function, pre and post-transplant diabetes, and pre-transplant hypertension. ^$^ Adjusted for age at transplant, pre- and post-transplant diabetes, pre-transplant hypertension. Ref: Reference group.

**Table 3:** Hazard ratios for graft survival based on achieving and/or maintaining optimal tacrolimus concentrations.

Risk Factors	Unadjusted			Adjusted ^#^		
	Number at Risk	HR (95% CI)	*p*-Value	Number at Risk	HR (95% CI)	*p*-Value
1-year graft survival						
$TAC achieved in 1st month YES (Ref: not achieved)	*n* = 159	1.63 (0.48–5.56)	0.435	*n* = 146	1.45 (0.41–5.11)	0.566
Age at transplant	*n* = 184	1.04 (0.99–1.08)	0.103		1.04 (0.99–1.09)	0.119
$HB target achieved at month 1	*n* = 161	0.37 (0.15–0.93)	0.035		.0.39 (0.14–1.10)	0.077
$TAC maintained for 12 months YES (Ref: not maintained)	*n* = 140	2.09 (0.27–16.11)	0.477	*n* = 136	2.31 (0.30–18.04)	0.425
Age at transplant	*n* = 184	1.04 (0.99–1.08)	0.103		1.05 (0.99–1.11)	0.127
$HB maintained for 12 months YES (Ref: not maintained)	*n* = 164	0.29 (0.10–0.80)	0.018		0.73 (0.19–2.77)	0.646
$Co-medications in preceding 12 months YES	*n* = 179	0.19 (0.03–1.40)	0.103		0.37 (0.05–2.90)	0.346
2-year graft survival						
$TAC maintained for 24 months YES (Ref: not maintained)	*n* = 148	1.55 (0.46–5.17)	0.478	*n* = 144	1.66 (0.49–5.64)	0.417
Age at transplant	*n* = 184	1.04 (1.00–1.07)	0.034		1.05 (1.00–1.10)	0.030
$HB maintained for 24 months YES (Ref: not maintained)	*n* = 166	0.30 (0.14–0.63)	0.002		0.52 (0.21–1.27)	0.148
$Co-medications in preceding 24 months YES	*n* = 179	0.12 (0.02–0.89)	0.038		0.22 (0.03–1.62)	0.136
3-year graft survival						
$TAC maintained for 36 months YES (Ref: not maintained)	*n* = 149	0.69 (0.28–1.69)	0.420	*n* = 145	0.71 (0.29–1.77)	0.464
Age at transplant	*n* = 184	1.04 (1.00–1.07)	0.020		1.04 (1.00–1.08)	0.044
$HB maintained for 36 months YES (Ref: not maintained)	*n* = 166	0.17 (0.09–0.34)	<0.001		0.31 (0.14–0.69)	0.004
$Co-medications in preceding 36 months YES	*n*= 179	0.09 (0.01–0.69)	0.020		0.18 (0.02–1.37)	0.098
4-year graft survival						
$TAC maintained for 48 months YES (Ref: not maintained)	151	0.83 (0.35–2.00)	0.682	*n* = 149	0.87 (0.36–2.11)	0.757
Age at transplant	184	1.04 (1.01–1.07)	0.011		1.04 (1.00–1.07)	0.032
$HB maintained for 48 months YES (Ref: not maintained)	168	0.21 (0.11–0.41)	<0.001		0.29 (0.14–0.59)	0.001
5-year graft survival						
$TAC maintained for 60 months YES (Ref: not maintained)	*n* = 151	0.75 (0.36–1.55)	0.432	*n* = 147	0.77 (0.37–1.62)	0.493
Age at transplant	*n* = 184	1.03 (1.01–1.06)	0.010		1.04 (1.01–1.07)	0.012
$HB maintained for 6 0 months YES (Ref: not maintained)	*n* = 168	0.17 (0.09–0.30)	<0.001		0.22 (0.12–0.43)	<0.001
Co-medications in preceding 60 months YES	*n* = 179	0.26 (0.08–0.85)	0.025		0.53 (0.16–1.78)	0.304

^#^ Adjusted. Ref: Reference group. $: TAC maintained: Maintenance of optimal concentration was considered when a trough level of 5–10 ng/mL was recorded in more than 33% of measurements. HB maintained: maintaining a haemoglobin level >10 g/dL (considered optimal) in more than 75% of measurements. Co-medications in preceding timepoints: co-medications (Verapamil/Diltiazem, Amlodipine, and/or Carvedilol) were taken concomitantly at more than 75% of measurements.

## Data Availability

The data presented in this study are available on request from the corresponding author due to reasons of sensitivity.

## Supplementary Materials

The following supporting information can be downloaded at: https://www.mdpi.com/article/10.3390/pharmaceutics18010132/s1, Supplementary Table S1: Median daily dose of immunosuppressant (tacrolimus); Supplementary Table S2: Median trough level of tacrolimus; Supplementary Table S3: Median dose and weight-adjusted tacrolimus trough levels (ng/mL/mg/kg); Supplementary Table S4: Approach to drug–drug interactions between tacrolimus and other concomitantly administered drugs; Supplementary Table S5: Comparison of biopsy diagnoses between recipients whose grafts survive and those who lost their graft in 5 years; STROBE Statement checklist.
