# Supplementary material for: Clinical Characteristics, Long-Term Pharmacokinetics, and Outcomes in Kidney Transplant Recipients from an African Tertiary Centre: A 10-Year Single-Centre Retrospective Review
Source: Pharmaceutics. 2026 Jan 21;18(1):132. doi: 10.3390/pharmaceutics18010132 (PMC12845319; doi:10.3390/pharmaceutics18010132)
Supplement: Supplementary file 1 [file pharmaceutics-18-00132-s001.zip › pharmaceutics-3919766-supplementary.pdf]

ARTICLE TITLE

Clinical Characteristics, Long-term Pharmacokinetics, and Outcomes in Kidney Transplant Recipients from an African Tertiary Centre: A 10-Year Single-Centre Retrospective Review.

Sadiq Aliyu Hussaini[1,2], Caroline Dickens[1], Confidence Makgoro[1], Therese Dix Peek[1], Badar Munir[1], Jeevan Perumala[1], Simran Patel[1], Qaiser Goolam[1], Graham Paget[1], Bala Waziri[1,3], Raquel Duarte[1].

1. **Department of Internal Medicine, Faculty of Health Sciences. University of the Witwatersrand, Johannesburg, South Africa.**
2. **Department of Clinical Pharmacology and Therapeutics, Ibrahim Badamasi Babangida University, Lapai. Nigeria.**
3. **Department of Medicine, Ibrahim Badamasi Babangida University, Lapai. Nigeria.**

Corresponding author: Sadiq Aliyu Hussaini.

Email: 1906480@students.wits.ac.za

Phone: +2348060142913. +27837181174

Supplementary Table S1. Median daily dose of immunosuppressant (tacrolimus).

**Median daily dose (mg/day)**							
	**Overall**		**5-year graft survival**		**Graft loss**		**p-value**
	n	Median (IQR)	n	Median (IQR)	n	Median (IQR)	
Day 7	151	10.0 (8.0 - 12.0)	97	9.9 (8.0 - 12.0)	54	10.0 (8.0 - 12.0)	0.712
Day 14	137	12.0 (8.0 - 14.0)	85	12.0 (8.0 - 14.0)	52	12.0 (9.5 - 15.5)	0.440
Month 1	142	12.0 (10.0 - 14.0)	93	12.0 (9.0 - 14.0)	49	12.0 (10.0 - 14)	0.426
Month 2	127	12.0 (8.0 - 14.0)	87	11.0 (8.0 - 14.0)	40	12.0 (10.0 - 14)	0.259
Month 3	123	10.0 (7.0 - 13.0)	87	10.0 (7.0 - 13.0)	36	10.5 (7.5 - 13.5)	0.927
Month 6	121	8.0 (6.0 - 10.0)	87	8.0 (6.0 - 10.0)	34	8.5 (6.0 - 12.0)	0.585
Month 9	118	8.0 (5.0 - 10.0)	86	8.0 (5.0 - 10.0)	32	10.0 (5.5 - 12.0)	0.110
Month 12	115	8.0 (5.0 - 10.0)	87	8.0 (5.0 - 10.0)	28	9.0 (6.0 - 12.0)	0.201
Month 18	119	7.0 (4.0 - 10.0)	92	7.0 (4.0 - 9.0)	27	8.0 (5.0 - 10.0)	0.446
Month 24	111	6.0 (4.0 - 8.0)	90	6.0 (4.0 - 8.0)	21	8.0 (5.0 - 10.0)	0.329
Month 30	102	6.0 (4.0 - 8.0)	87	6.0 (4.0 - 8.0)	15	8.0 (6.0 -11.0)	0.063
Month 36	101	6.0 (4.0 - 8.0)	87	6.0 (4.0 - 8.0)	14	6.0 (5.0 - 10.0)	0.241
Month 42	94	6.0 (4.0 - 9.0)	83	6.0 (4.0 - 8.0)	11	7.0 (4.0 - 10.0)	0.362
Month 48	90	6.0 (4.0 - 8.0)	83	6.0 (4.0 - 8.0)	7	5.0 (4.0 - 8.0)	0.940
Month 54	82	5.0 (4.0 - 8.0)	77	5.0 (4.0 - 8.0)	5	5.0 (4.0 – 8.0)	0.696
Month 60	75	5.0 (3.0 - 8.0)	75	5.0 (3.0 - 8.0)	0	-	-

Supplementary table S2. Median trough level of Tacrolimus.

**Median Tacrolimus trough level (ng/ml)**							
	**Overall**		**5-year graft survival**		**Graft loss**		**p-value**
	n	Median (IQR)	n	Median (IQR)	n	Median (IQR)	

Day 7	146	4.8 (2.9 - 6.4)	91	4.8 (3.1 - 6.3)	55	4.7 (2.8 – 6.9)	0.994
Day 14	128	5.3 (3.3 - 7.1)	76	5.3 (3.2 - 7.5)	52	5.2 (3.6 – 6.8)	0.614
Month 1	139	7.4 (5.4 - 10.2)	91	7.5 (5.5 - 10.3)	48	7.4 (4.7 - 9.9)	0.462
Month 2	123	7.6 (5.8 - 9.8)	83	8.0 (6.6 - 10.1)	40	7.0 (5.3 - 8.7)	**0.033**
Month 3	119	7.8 (6.2 – 10.0)	84	8.2 (6.6 - 10.0)	35	6.7 (5.3 - 9.1)	0.100
Month 6	117	6.1 (4.4 - 8.4)	84	6.7 (4.7 - 8.9)	33	5.1 (4.1 - 6.2)	**0.017**
Month 9	116	6.1 (4.4 - 7.8)	84	6.5 (4.6 - 8.0)	32	5.7 (4.0 - 6.5)	0.104
Month 12	113	5.7 (4.4 - 7.4)	86	5.7 (4.4 - 7.4)	27	5.5 (3.5 - 8.0)	0.512
Month 18	115	6.0 (4.7 - 8.2)	90	6.2 (4.7 - 8.3)	25	5.2 (4.6 - 7.6)	0.246
Month 24	107	6.1 (4.6 - 8.2)	87	6.1 (4.8 - 8.2)	20	5.9 (3.7 - 7.9)	0.479
Month 30	95	6.7 (4.1 - 7.7)	81	6.8 (4.3 - 7.6)	14	5.9 (3.6 - 8.3)	0.797
Month 36	98	6.2 (4.9 - 7.5)	85	6.1 (4.9 - 7.1)	13	6.5 (5.0 - 8.2)	0.519
Month 42	87	6.1 (4.5 - 7.8)	77	6.2 (4.8 - 7.8)	10	3.3 (2.6 – 4.5)	**0.006**
Month 48	77	5.7 (4.7 - 6.7)	72	5.7 (4.8 - 6.7)	5	5.2 (4.7 - 6.2)	0.495
Month 54	75	5.5 (4.1 - 7.0)	71	5.5 (4.1 - 7.0)	4	4.0 (1.9 - 7.5)	0.334
Month 60	74	4.9 (4.0 - 6.6)	74	4.9 (4.0 - 6.6)	-	-	-

Supplementary table S3. Median dose and weight-adjusted Tacrolimus trough levels (ng/ml/mg/kg).

**Median normalised Tacrolimus trough level (ng/ml/mg/kg)**							
	**Overall**		**5-year graft survival**		**Graft loss**		**p-value**
	n	Median (IQR)	n	Median (IQR)	n	Median (IQR)	
Day 7	123	28.8 (19.7 - 43.8)	85	28.6 (20.1 - 44.1)	38	30.4 (19.5 - 39.1)	0.726
Day 14	107	30.4 (20.8 - 44.2)	70	30.5 (21.5 - 43.3)	37	30.4 (18.5 - 45.7)	0.791
Month 1	124	44.5 (27.6 - 57.7)	89	43.5 (28.1 - 59.2)	35	45.4 (26.5 - 57.1)	0.879
Month 2	115	48.7 (30.7 – 69.3)	82	51.3 (32.5 - 77.3)	33	38.8 (26.3 - 63.0)	0.139

Month 3	112	53.6 (36.3 - 77.4)	82	54.6 (38.7 - 76.4)	30	43.8 (30.1 – 77.6)	0.284
Month 6	113	52.5 (33.6 - 79.1)	83	55.0 (33.6 - 79.1)	30	39.1 (26.4 - 71.1)	**0.040**
Month 9	113	55.6 (37.3 – 86.4)	83	61.2 (42.0 - 91.5)	30	48. (31.0 - 75.7)	0.101
Month 12	112	59.2 (37.6 – 85.1)	85	60.1 (41.1 - 92.8)	27	56.0 (27.5 – 73.1)	0.101
Month 18	112	69.0 (41.3 – 89.4)	88	72.4 (44.7 – 101.6)	24	53.2 (27.4 - 73.8)	**0.012**
Month 24	104	74.4 (43.5 - 102.7)	86	74.4 (50.0 - 107.6)	18	71.2 (31.2 - 84.5)	0.109
Month 30	92	76.3 (44.6 - 105.3)	79	79.0 (44.8 - 106.4)	13	58.8 (26.4 – 78.6)	0.079
Month 36	95	73.8 (48.1 - 116.1)	83	79.0 (48.5 – 125.4)	12	59.3 (40.1 – 76.0)	0.158
Month 42	84	75.1 (45.7 - 132.8)	76	78.3 (50.4 - 135.7)	8	36.9 (19.1 - 59.3)	**0.003**
Month 48	76	69.3 (46.8 – 122.0)	71	72.0 (46.9 - 127.5)	5	62.8 (42.7 – 66.7)	0.190
Month 54	73	74.2 (52.3 - 117.5)	69	76.8 (55.9 - 117.5)	4	45.6 (31.9 – 94.9)	0.208
Month 60	72	70.6 (46.1 – 104.3)	72	70.6 (46.1 – 104.3)	-	-	-

Supplementary Table S4: approach drug-drug interactions between tacrolimus and other concomitantly administered drugs.

Tacrolimus concentration level not recorded (a).		Tacrolimus concentration level was recorded and **not** marked (b).		Tacrolimus concentration level recorded and marked (c).
AMIODARONE	CLARITHROMYCIN	ISONIAZID	METRONIDAZOLE	DILTIAZEM
CARBAMAZEPINE	ERYTHROMYCIN	RIFAMPICIN	IBUPROFEN	VERAPAMIL
PHENOBARBITAL		RIFABUTIN	DICLOFENAC	AMLODIPINE
MEPHENYTOIN		EFAVIRENZ	INDOMETHACIN	CARVEDILOL
CLOTRIMAZOLE		NEVIRAPINE	FLUOXETINE	
FLUCONAZOLE		ACE - INHIBITORS	SERTRALINE	
ITRACONAZOLE		PROTON PUMP INHIBITORS	HALOPERIDOL	
KETOCONAZOLE		CIMETIDINE		
VORICONAZOLE		ANTACIDS		
MICONAZOLE		NIFEDIPINE		
AMPHOTERICIN B		NICARDIPINE		
ALL PROTEASE INHIBITORS		PROPRANOLOL		
ACYCLOVIR		LEVOFLOXACIN		

(a): Tacrolimus whole blood concentration level measurements while recipients are on these drugs were omitted during data collection.

(b): Tacrolimus whole blood concentration level measurements while recipients are on these drugs were recorded but not specifically marked as such during data collection.

(c): Tacrolimus whole blood concentration level measurements while recipients are on these drugs were recorded and marked as such during data collection.

Supplementary Table S5. Comparison of biopsy diagnoses between recipients whose grafts survive and those who lost their graft in 5 years.

Diagnosis	Graft loss (55)	Graft Survive (91)	p-value
>0 Rejection	22 (40.0%)	15 (16.5%)	0.0030
1 rejection	15 (27.3%)	12 (13.2%)	0.0569
2 rejections	5 (9.1%)	3 (3.3%)	0.1534
3 Rejections	2 (3.6%)	0	0.1403
BK virus nephropathy	5 (9.1%)	3 (3.3%)	0.1534
Nephrotoxicity	10 (18.2%)	16 (17.6%)	1.0000
Rejection + Nephrotoxicity	5 (9.1%)	1 (1.1%)	0.0285
Nephrotoxicity + BK virus nephropathy + Rejection	1 (1.8%)	0	0.3767
At least one of Rejection, Nephrotoxicity, or BK virus nephropathy	33 (60.0%)	33 (36.3%)	0.0088

P-values calculated using chi-square/Fisher's exact test.

STROBE Statement—Checklist of items that should be included in reports of ***cohort studies***

	Item No	Recommendation	Page No
**Title and abstract**	1	(*a*) Indicate the study's design with a commonly used term in the title or the abstract<br>(*b*) Provide in the abstract an informative and balanced summary of what was done and what was found	
**Introduction**			
Background/rationale	2	Explain the scientific background and rationale for the investigation being reported	
Objectives	3	State specific objectives, including any prespecified hypotheses	
**Methods**			
Study design	4	Present key elements of study design early in the paper	
Setting	5	Describe the setting, locations, and relevant dates, including periods of recruitment, exposure, follow-up, and data collection	
Participants	6	(*a*) Give the eligibility criteria, and the sources and methods of selection of participants. Describe methods of follow-up<br>(*b*) For matched studies, give matching criteria and number of exposed and unexposed	

Variables	7	Clearly define all outcomes, exposures, predictors, potential confounders, and effect modifiers. Give diagnostic criteria, if applicable	
Data sources/ measurement	8*	For each variable of interest, give sources of data and details of methods of assessment (measurement). Describe comparability of assessment methods if there is more than one group	
Bias	9	Describe any efforts to address potential sources of bias	
Study size	10	Explain how the study size was arrived at	
Quantitative variables	11	Explain how quantitative variables were handled in the analyses. If applicable, describe which groupings were chosen and why	
Statistical methods	12	(*a*) Describe all statistical methods, including those used to control for confounding<br>(*b*) Describe any methods used to examine subgroups and interactions<br>(*c*) Explain how missing data were addressed<br>(*d*) If applicable, explain how loss to follow-up was addressed<br>(*e*) Describe any sensitivity analyses	
**Results**			
Participants	13*	(a) Report numbers of individuals at each stage of study—eg numbers potentially eligible, examined for eligibility, confirmed eligible, included in the study, completing follow-up, and analysed<br>(b) Give reasons for non-participation at each stage<br>(c) Consider use of a flow diagram	
Descriptive data	14*	(a) Give characteristics of study participants (eg demographic, clinical, social) and information on exposures and potential confounders<br>(b) Indicate number of participants with missing data for each variable of interest<br>(c) Summarise follow-up time (eg, average and total amount)	
Outcome data	15*	Report numbers of outcome events or summary measures over time	

Main results	16	(*a*) Give unadjusted estimates and, if applicable, confounder-adjusted estimates and their precision (eg, 95% confidence interval). Make clear which confounders were adjusted for and why they were included<br>(*b*) Report category boundaries when continuous variables were categorized<br>(*c*) If relevant, consider translating estimates of relative risk into absolute risk for a meaningful time period	
Other analyses	17	Report other analyses done—eg analyses of subgroups and interactions, and sensitivity analyses	
**Discussion**			
Key results	18	Summarise key results with reference to study objectives	
Limitations	19	Discuss limitations of the study, taking into account sources of potential bias or imprecision. Discuss both direction and magnitude of any potential bias	
Interpretation	20	Give a cautious overall interpretation of results considering objectives, limitations, multiplicity of analyses, results from similar studies, and other relevant evidence	
Generalisability	21	Discuss the generalisability (external validity) of the study results	
**Other information**			
Funding	22	Give the source of funding and the role of the funders for the present study and, if applicable, for the original study on which the present article is based	

*Give information separately for exposed and unexposed groups.

Note: An Explanation and Elaboration article discusses each checklist item and gives methodological background and published examples of transparent reporting. The STROBE checklist is best used in conjunction with this article (freely available on the Web sites of PLoS Medicine at http://www.plosmedicine.org/, Annals of Internal Medicine at http://www.annals.org/, and Epidemiology at http://www.epidem.com/). Information on the STROBE Initiative is available at http://www.strobe-statement.org.
